# Readiness of and barriers for community pharmacy professionals in providing and implementing vaccination services

**DOI:** 10.1186/s12913-024-11349-6

**Published:** 2024-07-30

**Authors:** Wondim Ayenew, Yeniewa Kerie Anagaw, Liknaw Workie Limenh, Wudneh Simegn, Gizachew Kassahun Bizuneh, Teshome Bitew, Tefera Minwagaw, Ayelign Eshete Fitigu, Misganaw Gashaw Dessie, Getahun Asmamaw

**Affiliations:** 1 Department of Social and Administrative Pharmacy, School of Pharmacy, College of Medicine and Health Sciences, University of Gondar, Gondar, Ethiopia; 2 Department of Pharmaceutical Chemistry, School of Pharmacy, College of Medicine and Health Sciences, University of Gondar, Gondar, Ethiopia; 3 Department of Pharmaceutics, School of Pharmacy, College of Medicine and Health Sciences, University of Gondar, Gondar, Ethiopia; 4 Department of Pharmacognosy, School of Pharmacy, College of Medicine and Health Sciences, University of Gondar, Gondar, Ethiopia; 5 Department of Pharmacy, Pawi Health Science College, Pawi, Ethiopia; 6 Department of Pharmacy, College of Medicine and Health Sciences, Bahir Dar University, Bahir Dar, Ethiopia; 7 Department of Pharmacy, College of Medicine and Health Sciences, Debre Markos University, Arba Minch, Ethiopia; 8 Department of Pharmacy, College of Medicine and Health Sciences, Arba Minch University, Arba Minch, Ethiopia

**Keywords:** Community pharmacy, Vaccination services, Perception, Barriers, Implementation, Ethiopia

## Abstract

**Background:**

Community pharmacy professionals are essential for healthcare delivery, particularly for administering vaccination services. However, there is a lack of substantial evidence documenting their role in vaccination within Ethiopia.

**Objectives:**

This study aimed to assess community pharmacy professionals’ readiness to provide vaccination services, identify barriers hindering the implementation of these services, and determine factors influencing the provision of vaccination services by community pharmacy professionals.

**Methods:**

A cross-sectional study was conducted among community pharmacy professionals in Debre Markos and Injibara Town from April 15 to May 13, 2024. The data were collected using a structured questionnaire, and descriptive statistics were used to analyze the findings.

**Results:**

The study revealed that a significant majority of community pharmacy professionals perceived that they had adequate vaccine knowledge and were easily accessible to the community. However, barriers such as lack of regulation, time constraints, workload concerns, patient trust issues, and infrastructure challenges hinder the implementation of vaccination services. Factors influencing the provision of vaccination services included the need for enhanced education and training, financial reimbursement, patient demand, infrastructure improvements, collaboration with other healthcare providers, and pharmacists’ special interest in vaccination.

**Conclusions:**

Community pharmacy professionals exhibit readiness to provide vaccination services. However, significant barriers such as regulatory constraints, time pressures, workload concerns, patient trust issues, and infrastructure challenges hinder their full participation. Addressing these barriers and leveraging pharmacists’ expertise is essential for optimizing service delivery and improving public health outcomes. Advocating for policy changes, developing comprehensive training programs, establishing clear guidelines, investing in infrastructure improvements, conducting public awareness campaigns, and fostering collaboration with other healthcare providers are recommended to facilitate the provision and implementation of vaccination services by community pharmacy professionals in Ethiopia.

## Introduction

Vaccination is advised at all stages of life to prevent vaccine-preventable diseases and their complications, serving as a critical component of global communicable disease control efforts [1]. Community pharmacy professionals have become pivotal in delivering vaccination services, significantly contributing to public health outcomes. This shift has been driven by factors such as the accessibility of pharmacies and patients’ trust in pharmacists [2]. Globally, policies have expanded the scope of practice to include vaccine administration [3]. Pharmacists are motivated to enhance public health by increasing vaccination rates and preventing disease outbreaks [4]. Studies have shown that pharmacies are often more accessible than other healthcare facilities, especially in underserved areas, making them ideal locations for administering vaccines [5]. The convenience of receiving vaccinations at local pharmacies can also increase patient compliance [6].

In many developed countries, such as the United States [7], the United Kingdom [8], and Canada [9], community pharmacy professionals offer immunization services to enhance accessibility. The success of pharmacist-led vaccination programs in these nations indicates that pharmacists can significantly improve vaccination coverage in low- and middle-income countries (LMICs) (8–9). Community pharmacy professionals in various settings, such as Saudi Arabia [10], the United Arab Emirates [11] and Jordan [12, 13], have demonstrated readiness to lead pharmacy-based vaccination services.

However, limited evidence exists regarding the current involvement of pharmacists in vaccination services in LMICs [14–16]. Existing studies have revealed varying levels of knowledge and attitudes among community pharmacy professionals regarding vaccination services [17–19]. In Saudi Arabia, a significant proportion of pharmacists exhibit poor knowledge about vaccines [17]. Conversely, in Lebanon, more than 90% of pharmacists have good knowledge and positive attitudes toward providing vaccination services [18]. Moreover, in Malaysia, studies have reported that approximately half of pharmacists possess moderate knowledge levels [19].

Despite community pharmacy professionals’ readiness to be involved in vaccination services, significant barriers hinder community pharmacy professionals from providing vaccination services. The most significant barrier is the absence of authorization, followed by worries about expenses and time commitment required for professional development and training [20]. Pharmacy professionals also face logistical issues, such as insufficient storage space for vaccinations and store vaccines properly [21]. Financial constraints, such as inadequate reimbursement rates for vaccine administration, discourage pharmacy professionals from offering these services [22]. The administrative burden of maintaining records and reporting to vaccination registries is another significant deterrent [23]. Public perception and trust also play crucial roles. Although pharmacy professionals are trusted healthcare professionals, some patients may prefer vaccinations from doctors or nurses [24].

In Ethiopia, there is a growing interest among community pharmacy professionals in expanding their roles to improve public health outcomes [25]. Despite this interest, limited research has been conducted on their involvement in vaccination services. However, significant barriers must be addressed to enable their effective participation as vaccinators [20]. Addressing these barriers requires targeted strategies, including enhanced training programs, financial incentives, and public awareness campaigns, to highlight pharmacy professionals’ role in vaccination [26]. Therefore, this study aimed to explore the willingness of community pharmacists to provide vaccination services and identify obstacles hindering their full participation in this crucial public health initiative.

## Methodology

### Study setting and period

The study was conducted in the towns of Debre Markos and Injibara. Debre Markos is located 299 km from Addis Ababa. Indiana, the administrative center of the Awi Zone in the Amhara Region, is situated 447 km from Addis Ababa, the capital city. Debre Markos town had 15 community pharmacies and 20 drug stores, whereas Injibara town had 7 community pharmacies and 25 drug stores. The data were collected between April 15 to May 13, 2024.

### Study design

This study employed a cross-sectional design and was conducted among community pharmacy professionals in Debre Markos and Injibara town.

### Population

#### Study population

The study population consisted of community pharmacy professionals working in Debre Markos and Injibara Towns.

#### Source population

The source population included community pharmacy professionals registered with the Ethiopian Food and Drug Authority (EFDA) who worked in community pharmacies and drug stores at Debre Markos and Injibara Town.

#### Inclusion and exclusion criteria

The study included licensed community pharmacy professionals currently employed in community pharmacies and drug stores in Debre Markos and Injibara Town who had been practicing in their current roles for at least six months and who were willing to participate after providing informed consent. Community pharmacy professionals not actively practicing in a community pharmacy or drug store setting, as well as those employed in hospital or clinical settings who do not engage in community pharmacy practice, were excluded from the study.

#### Sampling

The study used a census sampling technique, as it included all actively serving pharmacy professionals from the two towns of Debre Markos and Injibara. The questionnaires were distributed to community pharmacy professionals present and available at their workplace during the study period. This approach ensured comprehensive coverage and provided a complete and accurate assessment of the research topic by encompassing the entire population of community pharmacy professionals in the target locations.

### Data collection procedure

The data were collected through a structured self-administered questionnaire. The questionnaire collected quantitative data on the extent of the vaccination services provided. The questionnaire was adapted from a previously published study [10]. Prior to the main study, a questionnaire draft was piloted with 5 pharmacy professionals outside the study area to evaluate comprehension, design, and length. Based on the feedback from this pretest, minor adjustments were made to certain parts of the questionnaire to ensure that it was easily understandable to the respondents.

The questionnaire used in this study comprises four distinct sections. First, the sociodemographic information section consists of eight questions aimed at gathering data regarding the demographic characteristics of community pharmacists participating in the research. Second, six questions were asked about the reasons for community pharmacy professionals providing vaccination services. This part of the questionnaire sought to explore the motivating factors behind community pharmacy professionals offering vaccination services.

The barriers affecting community pharmacy professionals’ willingness to provide vaccination services comprised 13 questions. Here, the focus is on identifying obstacles that hinder community pharmacy professionals’ full engagement in providing vaccination services. Finally, the community pharmacy professionals’ responses to the elements needed to implement the vaccination services section included ten questions.

Data collection was conducted by two trained pharmacists. During the study period, 57 questionnaires were distributed to community pharmacy professionals. In the end, data were collected from 46 respondents, resulting in a response rate of 80.7%.

### Operational definitions

A pharmacist is a registered and licensed pharmacy professional with a bachelor’s degree in pharmacy.

A pharmacy technician is a registered and licensed pharmacy professional with a 3-year diploma.

Community pharmacy professionals include pharmacists and pharmacy technicians who collaborate to provide essential medication dispensing, patient counseling, and health education services to the community.

### Data analysis

Frequencies and percentages were calculated for categorical variables, and medians and interquartile ranges were calculated for continuous variables that were not normally distributed. STATA 17 statistical software was used for data analysis.

## Results

### Sociodemographic Information about the respondents

The majority of the participants (54.35%) were female, while 45.65% were male. Marital status indicates a predominantly married population (56.52%), followed by singles (41.30%) and a smaller divorced segment (2.17%). The median age of the respondents was 31 years (interquartile range (IQR) of 24 to 37 years. Approximately 54.35% of respondents had a bachelor’s degree, and 45.65% had a diploma. In contrast to public institutions (36.96%), private institutions account for the majority of graduates (63.04%). The median work experience of both pharmacists and community pharmacists was 3 years, with IQRs of [1–8] and [1–5], respectively. In terms of roles, pharmacists accounted for 43.48% of the sample, while technicians comprised 56.52% (Table 1).

**Table 1:** Sociodemographic information of the respondents

Variable	Category	Frequency	Percentage
Gender	Female	25	54.35
	Male	21	45.65
Marital Status	Single	19	41.30
	Married	26	56.52
	Divorced	1	2.17
Age (in years) median + IQR 31+ [24–37]			
Education	Bachelor’s	25	54.35
	Diploma	21	45.65
Institute of Graduate	Public	17	36.96
	Private	29	63.04
Work experience as a pharmacist (in years) (median + IQR 3+ [1–8]			
Work experience as a community pharmacist (in years) (median + IQR 3+ [1–5]			
Position in community pharmacy	Pharmacist	20	43.48
	Technician	26	56.52

### Reasons for community pharmacists to provide vaccination services

The results of the present study indicated that a significant majority (58.70%) agreed that pharmacists possess adequate knowledge of vaccines. Accessibility was another key aspect, with 47.83% of respondents strongly agreeing and agreeing (39.13%) that pharmacists are easily accessible to the community. Regarding the impact of pharmacy-based vaccinations, 36.96% believe it will enhance the overall vaccination rate, while 41.30% see it positively affecting vaccination coverage rates. However, opinions on cost-effectiveness were divided, with equal proportions (28.26%) expressing agreement, disagreement, and neutrality. Concerning the role of pharmacists in vaccine promotion, 54.35% strongly agreed, highlighting their potential in advertising and advocating for vaccination (Table 2).

**Table 2:** Reasons for community pharmacists providing vaccination services

S.*N*	Variables	SD *n* (%)	D *n* (%)	NA *n* (%)	A *n* (%)	SA *n* (%)
1	Community pharmacists have good knowledge about vaccines and their indications	6 (13.04%)	1 (2.17%)	7 (15.22%)	27 (58.70%)	5 (10.87%)
2	Community pharmacists are easily accessible in the community	3 (6.52%)	0 (0.00%)	3 (6.52%)	18 (39.13%)	22 (47.83%)
3	Providing vaccinations through community pharmacy will improve the rate of vaccination in general	4 (8.70%)	4 (8.70%)	6 (13.04%)	17 (36.96%)	15 (32.61%)
4	Providing vaccination through a community pharmacy improves the vaccination rate (vaccination coverage rate)	1 (2.17%)	0 (0.00%)	9 (19.57%)	17 (36.96%)	19 (41.30%)
5	Cost effectiveness	6 (13.04%)	1 (2.17%)	13 (28.26%)	13 (28.26%)	13 (28.26%)
6	Community pharmacists can play an important role in advertising and promotion of vaccination	3 (6.52%)	1 (2.17%)	17 (36.96%)	25 (54.35%)	0 (0.00%)

### Barriers affecting community pharmacists in providing vaccination services

Approximately 54.35% of the pharmacists felt that they were busy and lacked sufficient time to provide vaccination services, indicating a potential barrier to service delivery. Additionally, 43.48% of respondents expressed concerns that adding more work in the form of vaccination services would further the burden on them. Patient safety emerged as a key concern, with over half of the respondents indicating that patients trust pharmacists less to provide such services, suggesting potential trust issues that need to be addressed. Furthermore, 34.78% felt uncomfortable using needles, which could have affected their willingness to administer vaccinations. Challenges related to infrastructure were also evident, with many pharmacists highlighting issues such as a lack of training (32.61%) and space for storage (52.17%), as well as inadequate reimbursement (41.30%) (Table 3).

**Table 3:** Barriers to community pharmacists’ vaccination services

S.*N*	Variables	SD *n* (%)	D *n* (%)	NA *n* (%)	A *n* (%)	SA *n* (%)
1	Pharmacists are busy and have no time to provide vaccination	12 (26.09)	25 (54.35)	9 (19.57)	0 (0.00%)	0 (0.00%)
2	Will add more work	9 (19.57)	20 (43.48)	5 (10.87)	11 (23.91)	1 (2.17)
3	Patient safety is a concern	4 (8.70)	14 (30.43)	8 (17.39)	12 (26.09)	8 (17.39)
4	Lack of training	2 (4.35)	11 (23.91)	7 (15.22)	15 (32.61)	11 (23.91)
5	Pharmacists are less trusted by patients when providing such services	4 (8.70)	23 (50.00)	0 (0.00%)	0 (0.00%)	15 (32.61)
6	The pharmacies are not designed (authorized) to accommodate such services.	9 (19.57)	10 (21.74)	11 (23.91)	15 (32.61)	1 (2.17)
7	Conflicts with other professionals who are eligible to be vaccinated	3 (6.52)	13 (28.26)	14 (30.43)	7 (15.22)	9 (19.57)
8	Concerns about handling vaccines, storage, and disposal of sharps	7 (15.22)	11 (23.91)	8 (17.39)	7 (15.22)	13 (28.26)
9	Pharmacists are not comfortable using needles	4 (8.70)	16 (34.78)	13 (28.26)	9 (19.57)	4 (8.70)
10	Lack of storage space	5 (10.87)	24 (52.17)	5 (10.87)	6 (13.04)	6 (13.04)
11	Lack of private areas for vaccination	5 (10.87)	17 (36.96)	17 (36.96)	3 (6.52)	4 (8.70)
12	Lack of reimbursement (profitability)	5 (10.87)	19 (41.30)	7 (15.22)	9 (19.57)	6 (13.04)
13	Low patient demand	6 (13.04)	25 (54.35)	5 (10.87)	7 (15.22)	3 (6.52)

### Factors influencing the implementation of community pharmacist vaccination services

This study revealed several key factors influencing the implementation of vaccination services by pharmacists. There was agreement (52.17%) among respondents regarding the importance of providing more university education and training on vaccination administration for pharmacists, indicating a recognized need for enhanced professional development in this area. Continuous education and training workshops were deemed crucial by a significant portion (39.13%) of pharmacists, highlighting the importance of ongoing skill development. Financial reimbursement emerged as another critical factor, with 41.30% of respondents emphasizing the need for adequate remuneration for pharmacists providing vaccination services. Moreover, there is acknowledgment of patients’ demand for vaccination services (43.48%), suggesting a potential driver for expanding these services. Practical considerations, such as providing specific areas within pharmacies for vaccination (47.83%) and relieving pharmacists from technical tasks (52.17%), are also highlighted as essential for facilitating service delivery. Collaboration with medical clinics (41.30%) and support from medical and nursing associations (56.52%) were identified as valuable strategies for enhancing vaccination services. This study revealed the significance of leveraging pharmacists’ specialty interest (69.57%) in vaccination, indicating a potential avenue for optimizing service delivery through targeted expertise. The majority (69.56%, strongly agreed with the statement that “there are no regulations allowing community pharmacists to provide vaccination services”, clearly indicating that most community pharmacists are aware of and acknowledge the lack of regulatory support for them to provide vaccination services (Table 4).

**Table 4:** Factors influencing the implementation of community pharmacist-based vaccination services

S.*N*	Variables	SD *n* (%)	D *n* (%)	NA *n* (%)	A *n* (%)	SA *n* (%)
1	More university education and training on vaccination administration among pharmacists	2 (4.35)	9 (19.57)	11 (23.91)	24 (52.17)	0 (0.00%)
2	Continuous education and training workshops on vaccination (increased training on vaccination and handling of vaccines)	3 (6.52)	3 (6.52)	7 (15.22)	18 (39.13)	15 (32.61)
3	Financial reimbursement or adequate payment	5 (10.87)	4 (8.70)	8 (17.39)	19 (41.30)	10 (21.74)
4	Patients’ demand	7 (15.22)	3 (6.52)	10 (21.74)	20 (43.48)	6 (13.04)
5	Provide a specific area for vaccination in the pharmacy	5 (10.87)	5 (10.87)	8 (17.39)	22 (47.83)	6 (13.04)
6	Relieved the technical tasks of pharmacists	2 (4.35)	7 (15.22)	9 (19.57)	24 (52.17)	4 (8.70)
7	Collaboration with medical clinics	2 (4.35)	11 (23.91)	9 (19.57)	19 (41.30)	5 (10.87)
8	Medical and nursing association support	1 (2.17)	3 (6.52)	12 (26.09)	26 (56.52)	4 (8.70)
9	Pharmacist specialty interest	4 (8.70)	0 (0.00%)	6 (13.04)	32 (69.57)	4 (8.70)
10	There are no regulations allowing community pharmacists to provide vaccination services	0 (0.00%)	0 (0.00%)	2 (4.35)	12 (26.09)	32 (69.56)

## Discussion

Most pharmacy professionals believe that they possess adequate knowledge of vaccines and that they consider themselves easily accessible to the community. This confidence in their expertise and accessibility aligns with trends observed in various countries worldwide, where pharmacists increasingly play crucial roles in public health initiatives, including vaccination programs [27]. Recent national policies and regulations have also increasingly involved community pharmacists in vaccination services in different countries, significantly enhancing public health initiatives. In the U.S., the Advisory Committee on Immunization Practices (ACIP) has included the American Pharmacists Association (APhA) in adult immunization schedule approval, with expanded authority for pharmacists to administer vaccines across all states [28, 29]. The UK has integrated community pharmacists into NHS vaccination programs, allowing them to deliver flu and COVID-19 vaccinations, supported by training programs to ensure competency [30].

Studies across different countries have also shown that pharmacists are generally perceived as accessible healthcare providers with adequate vaccine knowledge [3, 31]. A study conducted in the United States revealed that pharmacists are considered accessible and knowledgeable about vaccines, which is consistent with the findings of our study [21].

The cost-effectiveness of pharmacist-provided vaccination services differ across various healthcare systems and reimbursement models. Some studies have indicated that pharmacist involvement in vaccination can be cost-effective, especially where pharmacists can charge for their services [23]. However, other studies have raised concerns about cost-effectiveness, particularly in situations where reimbursement mechanisms are ambiguous or inadequate [32]. Additionally, there is evidence suggesting that vaccinations administered through community pharmacies are more cost-effective than those administered through medical settings [33].

In this study, despite community pharmacy professionals being confident, concerns were noted regarding barriers such as lack of regulation, time constraints, workload issues, patient trust, and infrastructure challenges. These obstacles have been consistently highlighted in studies conducted in various countries. For instance, a study in Canada revealed that pharmacists encounter similar barriers, including workload and patient trust issues [34]. Similar challenges have been identified in studies from Canada and the UAE [6, 35]. These findings are also consistent with studies demonstrating the benefits of authorizing pharmacists to administer vaccines in community settings [31, 36].

However, many community pharmacists have reported encountering significant barriers to providing vaccines, including concerns about legal liability, insufficient personal resources, and inadequate training. Similarly, numerous other studies have identified legal liability as the most critical barrier to offering immunization services in community pharmacies [9, 37–39]. Conversely, another study from Jordan highlighted the lack of authorization as the primary obstacle for community pharmacies to provide vaccination services [40]. Thus, it is essential to review and update the regulations in Ethiopia concerning the authorization of pharmacists to administer vaccines.

Similarly, the issue of patient trust in pharmacists’ ability to provide vaccination services resonates with research conducted in Uganda and Ethiopia, where trust in pharmacists as healthcare providers remains a crucial factor influencing patients’ utilization of pharmacy services [41, 42]. Therefore, establishing and maintaining trust is essential for successful vaccination initiatives in these regions.

Challenges related to infrastructure, such as lack of training, space for storage, and inadequate reimbursement, are also prevalent among the respondents. This infrastructure challenges mirror obstacles faced in many developing countries, where limited resources and logistical constraints hinder the delivery of comprehensive immunization services through pharmacies [43].

Infrastructure challenges, including lack of training, insufficient storage space, and inadequate reimbursement, have been documented in studies across Nigeria and Sudan [43, 44]. Limited resources and logistical constraints often hinder the expansion of vaccination services through pharmacies in these countries, mirroring the findings of the surveyed pharmacists.

Our study is also consistent with a study conducted in Saudi Arabia that identified several limiting factors, such as inadequate training and concerns about maintaining patient safety, that affect the role of community pharmacy professionals in vaccination services [10]. These issues highlight the need for community pharmacy professionals to receive improved education and vaccination training.

Despite these barriers, there has been a consensus among pharmacists regarding the importance of enhancing education and training on immunization administration. This emphasis on professional development echoes global efforts to empower pharmacists with the necessary skills and knowledge to actively contribute to vaccination campaigns [45].

In the current study, education and training, financial reimbursement, patient demand, and collaboration with other healthcare providers were the factors that influenced the implementation of pharmacist-provided vaccination services. These factors have also been cited in studies from different countries. A study conducted in the UK emphasized the importance of training and collaboration with general practitioners for the successful implementation of pharmacist-led vaccination services [46].

The role of pharmacists in vaccination services is increasingly recognized globally, with many countries expanding their scope of practice to include vaccination administration [47–49]. This shift has been driven by various policy initiatives aimed at improving vaccination coverage and healthcare access. In this study, a significant majority (69.56%, strongly affirmed the statement that “there are no regulations permitting community pharmacists to offer vaccination services.” This finding is consistent with those of previous studies highlighting regulatory barriers faced by pharmacists in expanding their roles to include vaccination services [3, 4]. However, in the United States, pharmacists are authorized to administer vaccines under state-specific regulations, which has significantly improved access to immunization services [47].

From a curriculum perspective, pharmacy education has evolved to incorporate training in vaccine administration, storage and management to prepare pharmacists for their expanded roles. This includes hands-on training in injection techniques, patient counseling, and vaccine schedule adherence [48]. Policy frameworks supporting pharmacist-administered vaccinations often emphasize collaboration between pharmacists, healthcare providers, and public health agencies to ensure coordinated immunization efforts. These policies leverage the accessibility of community pharmacies and pharmacists’ expertise in medication management to enhance public health outcomes [49]. The present study has certain limitations. This study’s limitations include its potentially limited sample size, which affects the generalizability of the findings, and its reliance on self-reported data, which may introduce biases. The lack of comparative data with pharmacists from other healthcare settings and the cross-sectional design, which captures perceptions at a single point in time further constrain the study. Additionally, external factors such as regional policies and economic conditions that could influence pharmacists’ willingness and ability to provide vaccination services were not fully considered.

## Conclusions

Community pharmacy professionals exhibit readiness to provide vaccination services. However, significant barriers such as regulatory constraints, time pressures, workload concerns, patient trust issues, and infrastructure challenges hinder their full participation. To optimize their role, regulatory reforms are essential to authorize pharmacists for vaccination administration, accompanied by liability protection measures. Continuous education and training programs must be integrated into pharmacy curricula and offered for professional development to enhance pharmacist skills. Addressing infrastructure needs, establishing clear reimbursement mechanisms, fostering interprofessional collaboration and conducting public awareness campaigns are crucial steps for overcoming existing barriers and empowering pharmacists to play a more prominent role in public health through vaccination services.

## Data Availability

All the data generated or analyzed during this study are included in the manuscript.
